# Factors of interrupting chemotherapy in patients with Advanced Non-Small-Cell Lung Cancer

**DOI:** 10.1186/1756-0500-3-164

**Published:** 2010-06-10

**Authors:** Rhizlane Belbaraka, Olivier Trédan, Isabelle Ray-Coquard, Giselle Chvetzoff, Agathe Bajard, David Pérol, Nabil Ismaili, Mohammed Ismaili, Hassan Errihani, Thomas Bachelot, Paul Rebattu

**Affiliations:** 1 Department of Medical Oncology, Centre Léon-Bérard, 28 Rue Laennec, Lyon-69008, France; 2 Department of Medical Oncology, National Institute of Oncology, Rabat-10000, Morocco; 3 Department of Biostatistics, Centre Léon-Bérard, 28 Rue Laennec, Lyon-69008, France; 4 Department of Microbiology, Moulay Ismail University, Meknes-50000, Morocco

## Abstract

**Background:**

Little is known about prognosis of metastatic patients after receiving a first-line treatment and failure. Our group already showed in pre-treated patients enrolled in phase I clinical trials that a performance status (PS) > 2 and an LDH > 600 UI/L were independent prognostic factors. In this prospective study, which included 45 patients, we identified clinical and biological variables as outcome predictors in metastatic Non-Small Cell lung cancer after first line chemotherapy were identified.

**Findings:**

Forty-five patients that were previously treated for metastatic disease from 12/2000 to 11/2005 in the comprehensive cancer centre (Centre Léon Bérard). Clinical assessment and blood parameters were recorded and considered. Patient prognostic factors for overall survival (OS) with a 0.05-significance level in univariate analysis were entered in a multivariate Cox model for further analysis.

Patients' median age was 58.5 years (range: 37 - 76). Sixty two percent of the patients were PS = 0 or 1. After inclusion, nine patients received second-line (22.5%), and two received third-line chemotherapy (5%). Univariate analysis showed that the factors associated with reduced OS were: PS > 2, weight loss >10%, more than one line of chemotherapy treatment and abnormal blood parameters (hemoglobin (Hb), platelet and neutrophils counts). Multiple regression analysis confirmed that PS > 2 and abnormal hemoglobin were independent predictors for low overall survival. According to the presence of none (33%), 1 (37%) and 2 (30%) prognostic factors, median OS were 12, 5 and 2 months respectively.

**Conclusion:**

From this prospective study, both PS and anemia were found as independent determinants of survival, we found that both PS and anemia were independent determinants of survival. The combination of poor PS and anemia is an effective strategy to predict survival in the case of patients with metastatic NSCLC receiving further treatment after the first line.

## Findings

Prediction of survival in the case of advanced non-small cell lung cancer (NSCLC), when patients have already received first line treatment, is critical to the decision of subsequent treatments. Defining prognostic determinants of NSCLC may help physicians to improve their decisions making for both clinical trials and routine practice [1].

Many studies have reported predictive models for survival in metastatic cancers. These models integrated a combination of clinical and biological factors. Biological characteristics such as lactate dehydrogenase level (LDH) [4], lymphocyte count [2,3], or Interleukin 6 level [5,6] have been correlated with poor outcome in metastatic cancer.

In this study, we aimed to identify clinical and biological variables as outcome predictors for early death in locally advanced or metastatic NSCLC in patients treated beyond the first line chemotherapy failure.

## Patients and methods

The investigation was a prospective, observational, single-centre study.

Inclusion criteria were: patients > 18 years old with locally Advanced or metastatic NSCLC who were eligible for further chemotherapy after first line chemotherapy failure.

Exclusion criteria were: new patients who had not yet received any chemotherapy.

Written consent was obtained from each patient. The institutional ethics committee approved the study protocol before implementation, on March 17, 2000.

The primary objective of this study was to identify clinical and biological variables as prognostic factors for metastatic NSCLC patients after failure of first line chemotherapy.

These variables were collected at inclusion: age, gender, Eastern Cooperative Oncology Group (ECOG) PS, weight loss, number and sites of metastases, previous and current chemotherapy regimens. In addition to laboratory tests which included complete blood accounts (hemoglobin, neutrophils, lymphocytes), albumin, LDH and C-reactive protein (CRP) levels. According to our previous studies, the cutoff of each biological parameter was defined as follows: the albumin level +/- 38 g/l, the lymphocytes count +/- 700 μL [2,3], the LDH level +/- 600 U/L [4], and anemia was defined as hemoglobin level (Hb) less than 11.5 g/dL for female and less than 13.0 g/dL for male.

### Statistical considerations

Survival analysis: the primary outcome was overall survival, defined as the time from inclusion to death or to last follow-up. Survival distributions in prognostic groups were estimated by the Kaplan-Meier method.

Univariate and multivariate analysis: to evaluate the relationship between survival and baseline characteristics, all clinical and biological variables were included in univariate Cox proportional hazard regression models. The log-rank test was used to compare survival curves. Candidate prognostic factors for overall survival with a 0.05 level of significance in univariate analysis were entered in a multivariate Cox model.

## Results

### Patients' characteristics (Table 1)

**Table 1:** Patients' characteristics

Patients Characteristics	Number (Percent)
Total	45 (100)

Age, years	
Median	58.5
Range	34.7 - 76.9

Gender	
Male	30 (66.7)
Female	15 (33.3)

ECOG Performance Status	
Assessed by the physician	
0	4 (9.3)
1	23 (53.5)
>1	16 (37.2)
Self-assessed by the patient	
0	0 (0.0)
1	17 (37.8)
>1	28 (62.2)

Weight loss > 10%	6 (14.0)

Number of metastases	
1	8 (18.2)
2	21 (47.7)
>2	15 (34.1)

Site of metastases	
Bone	11 (24.4)
Liver	14 (31.1)
Lung	28 (62.2)
Brain	12 (26.7)

Previous treatment	
Adjuvant chemotherapy	4 (8.9)
Treatment completed	
First-line	29 (72.5)
Second-line	9 (22.5)
>Second-line	2 (5.0)

Treatment after inclusion	
Palliative chemotherapy	45 (100.0)

Between 2000 and 2005, 45 patients with metastatic NSCLC were included in the study. Their characteristics are summarized in table 1. Thirty patients were male (75%) and fifteen female (25%). Median age was 58.5 years (range 37-76). Sixty two percent had PS 0 -1. Only 6 patients (14%) had a weight loss of 10% or more in the previous six months.

All patients received platinum based first line chemotherapy. Prior to inclusion, 9 patients received second line treatment and two patients received third line chemotherapy treatment (single agent Docetaxel or Gemcitabine).

Biological variables are summarized in Table 2. All laboratory data were available for all patients. Majority of the patients presented anemia (65%), low albumin level (77.5%) and high CRP level (54%).

**Table 2:** Biological tests

Parameters	Number (Percent)
Hemoglobin	
Abnormal (<11.5 g/dL for female; <13.0 g/dL for male)	29 (64.4)

Absolute Neutrophil Count	
<2000/μL	1 (2.2)
2000-7500/μL	31 (68.9)
>7500/μL	13 (28.9)

Lymphocyte count	
<700/μL	20 (46.5)
700-1000/μL	10 (23.3)
≥1000/μL	13 (30.2)
Missing data	2

Platelet count	
<130 G/L	5 (11.1)
130-400 G/L	33 (73.3)
>400 G/L	7 (15.6)

Albumin	
<38 g/L	31 (77.5)
Missing data	5

LDH	
>600 U/L	10 (27.0)
Missing data	8

CRP	
≤10 mg/L	15 (45.5)
10-50 mg/L	8 (24.2)
>50 mg/L	10 (30.3)
Missing data	12

LDH: lactate dehydrogenase; CRP: C-reactive protein;

### Survival

Because almost all patients died (42/45), the Kaplan- Meier method have not been used to calculate the follow-up. To estimate it, we provide the median of the delays of survival for all patients, which was 3.1 months (range: 0.2 - 46.7 months).

Univariate analysis showed that six variables were correlated with poor outcome (p < 0.05).

As clinical parameters, we found PS > 1, weight loss (<10%) and more than one line of chemotherapy treatment. Anemia, platelet level < 130000 and IL6 > 8 pg/ml were also associated with poor clinical outcome. Table 3 summarized results of prognostic parameters using univariate and multivariate analysis.

**Table 3:** Prognostic parameters on univariate and multivariate analysis for lung cancer patients

	Univariate analysis			Multivariate analysis		
	**HR**	**95% CI**	**p**	**HR**	**95% CI**	**p**

**ECOG Performance Status assessed by the physician**						

0-1 >1	3.641	[1.743-7.607]	0.0006	3.279	[1.546-6.955]	0.0020

**Hemoglobin**						

Normal Abnormal†	2.941	[1.401-6.172]	0.0044	2.781	[1.290-5.997]	0.0091

**Platelet count (G/L)**						

≥130 <130	4.023	[1.512-10.703]	0.0053			NS

**Weight loss >10%**						

No Yes	2.633	[1.079-6.423]	0.0334			NS

**Number of metastases**						

≤2 >2	1.776	[0.913-3.453]	0.0904			NS

**Number of chemotherapy lines**						

≤1 >1			0.0305			NS

**Albumin (g/L)**						

≥38 <38	1.585	[0.722-3.481]	0.2510			-

**CRP (mg/L)**						

≤10 >10	1.499	[0.718-3.132]	0.2812			-

**Lymphocyte Count**						

≥700/μL <700/μL	1.297	[0.684-2.462]	0.4258			-

**Liver metastases**						

No Yes	0.809	[0.415-1.581]	0.5359			-

**Quality of life using 0-10 VAS**						

≥5 <5	0.841	[0.445-1.592]	0.5953			-

**Age (years)**						
≤60 >60	0.919	[0.495-1.710]	0.7907			-

**Previous adjuvant chemotherapy**						

No Yes	0.863	[0.265-2.816]	0.8074			-

LDH (U/L) ≤600 >600	1.079	[0.501-2.326]	0.8462			-

**Absolute Neutrophil Count**						

≥2 <2	1.190	[0.161-8.801]	0.8648			-

LDH: lactate dehydrogenase; CRP: C-reactive protein; VAS: visual analog scale. †<11.5 g/dL for female; <13.0 g/dL for male

**Figure 1:**
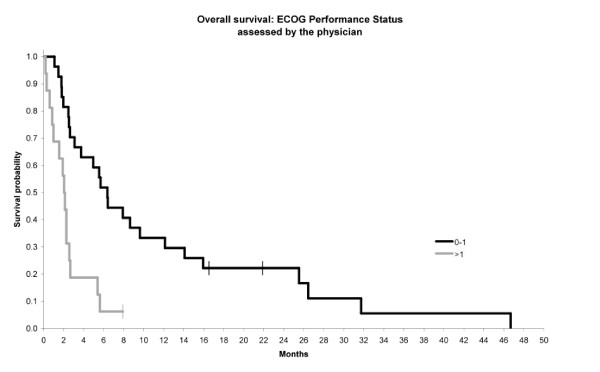
Relationship between performance status and overall survival

**Figure 2:**
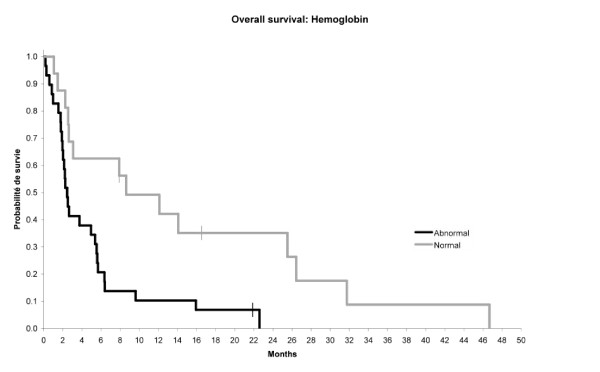
Relationship between hemoglobin level and overall survival

Multivariate analysis showed that only two variables were statistically correlated with significant poor prognosis (p < 0.05): PS > 1 and anemia. Figures 1 and 2 showed the relationship between PS or Hb level and overall survival. The median survival was 2 months when PS > 1 versus 8 months when PS: 0-1. The median survival was 3 months when Hb level was abnormal versus 9 months when Hb level was normal.

**Figure 3:**
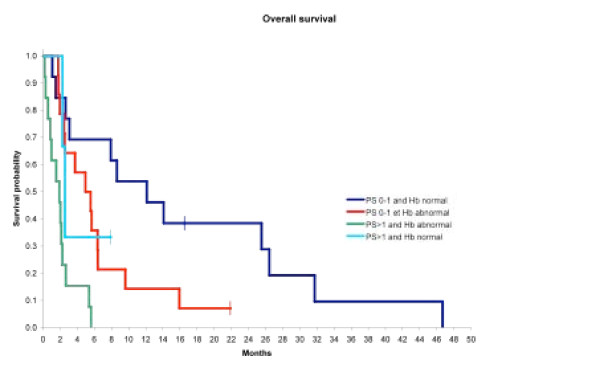
Results of analysis of 0, 1 or 2 prognostic factors with Kaplan-Meier overall survival analysis

We can then define 4 groups of survival. Figure 3 show the results of the analysis of 0, 1 or 2 prognostic factors with Kaplan-Meier overall survival analysis. Group1 (13 patients) was a poor outcome group with only 1.9 months median survival (CI95: 0.9-2.3) and had a PS > 1 and anemia.

Group 2 (13 patients) was a good outcome group with 12.1 months median survival (CI95: 3.1-26.4) and had a PS: 0-1 and normal Hb.

In addition, two intermediate groups were identified: one with a PS > 1 and normal Hb (3 patients) and had a median survival of 2.6 months (CI95: 2.3-NA), and a second with a PS: 0-1 and anemia (14 patients) and had a median survival of 5.3 months (CI95: 2.5-6.4).

## Discussion

From this prospective study, we found that both PS and anemia were independent prognostic factors of survival for metastatic NSCLC patients who relapsed after the first line chemotherapy. The combination of poor PS and anemia is an effective strategy to predict survival and then to select patients who may be will not benefit from further chemotherapy.

Up till now, most widely accepted prognostic determinants are disease stage and performance status [8]. Many large trials considered other features such as male gender, age older than 60 years, non-squamous histologics, weight loss as stratification factors insofar as they have been reported as negative prognostic factors [9].

As in other types of cancer, PS plays a clear prognostic role in advanced NSCLC. In all the retrospective and prospective trials regarding prognostic factors in this disease, PS has been shown to be an independent prognostic parameter [10].

For example, a series of 14 South Western Oncology Group (SWOG) trials, where 2531 patients with inoperable lung cancer were recruited during 1974 -1988 in order to investigate the impact that 50 prognostic factors had on survival, revealed that performance status, extent of disease, and weight loss were among the most important ones [11]. The median survival was found to be 6.7 months in PS 0,1 patients and 3. 8 months in PS 2 patients.

For patients with good PS, pretreatment Hb level >= 11 g/dl, normal LDH, calcium levels and a single metastatic site were favorable factors [11]. A recursive partitioning and amalgamation analysis showed that the best survival occurred in patients with good PS who had Hb levels >= 11 g/dl and were older than 47 years.

Further more, the Eastern Cooperative Oncology Group (ECOG) in a five phase II/III trials performed on 1960 patients with advanced NSCLC during 1981-1994 and treated with cisplatin-based chemotherapy showed a median survival of 9.4 months for patients with PS0, 6.4 months for patients with PS1, and 3.3 months for patients with PS2, confirming once again the prognostic value of performance status [12,13].

In our study, performance status (PS) was also identified as independent prognostic factor. Median survival was 8 months in PS: 0-1 patients, and 2 months in PS >1 patients.

Of all clinical and biological factors analyzed, PS and anemia were the only variables statistically significant in multivariate analysis. Neither presence of brain metastases nor a weight loss could predict patients' survival.

Anemia is commonly observed in lung cancer and is a major contributing factor to fatigue and decreased quality of life (QOL) [14]. Hemoglobin levels at the time of diagnosis can be considered as prognostic indicator in patients with NSCLC. Low hemoglobin levels are associated with decreased survival. The evaluation of this parameter can be used for a more accurate prognosis in this disease and a better adequacy of therapeutic indications [15].

Anemia is the most frequently observed haematological abnormality faced by cancer patients.

Yet, its association to tumour biology is not well understood. Several recent retrospective clinical studies showed that anemia is not only a negative prognostic factor but also, in some situations, a negative predictive parameter in chemotherapy-treated patients with solid tumours or haematological malignancies. These include lymphomas, leukaemias, non-small-cell lung cancer, ovarian cancer, cervical cancer, urothelial and renal cancers, and head and neck carcinoma [16,17].

The treatment of anemia in patients with cancer undergoing chemotherapy may improve outcome in terms of both response rate to treatment and survival.

Several clinical signs have been shown to be prognostically important in terminally ill cancer patients [18]. Symptoms like anorexia-cachexia syndrome or delirium and cognitive impairment are related with end-of-life and therefore are not relevant for patients undergoing chemotherapy. Other clinical signs, such as dyspnea and weight loss, have also proven effective to predict life expectancy. When choosing between chemotherapy and supportive care alone, patients' preferences should be taken into account, considering that most patients are willing to accept chemotherapy for a very small chance of benefit.

## Conclusion

The decision to stop chemotherapy is one of the hardest challenges in oncology practice. Chemotherapy remains widely prescribed for terminally ill patients despite side effects and poor efficacy.

From our prospective observational study, we concluded that factors influencing survival on patients with Advanced Non-Small-Cell lung cancer, who had at least one line of chemotherapy treatment, were anemia and poor PS. Larger prospective studies seem necessary to develop prognostic test scores, adapted to NSCLC, which may help clinicians estimate patients' survival and make appropriate recommendations for active or supportive care to develop prognostic scores, which may help clinicians estimate patients' survival and make appropriate recommendations for active or supportive care.

## List of abbreviations

NSCLC: non-small-cell lung cancer; PS: performance status; Hb: Heamoglobin;

## Competing interests

The authors declare that they have no competing interests.

## Authors' contributions

RB: Drafted the manuscript. OT: Participated in the design of the study, helped to draft the manuscript and review of the final manuscript and revising it critically for important intellectual content. OT and DP: Conceived of the study, and participated in its design and coordination. AB performed the statistical analysis. NI: Review of the final manuscript and revising it critically for important intellectual content. MI: English Writing. All authors read and approved the final manuscript.
